# Paclitaxel induces apoptosis through the TAK1–JNK activation pathway

**DOI:** 10.1002/2211-5463.12917

**Published:** 2020-07-31

**Authors:** Di Yu‐Wei, Zhuo‐sheng Li, Shu‐min Xiong, Ge Huang, Yan‐fei Luo, Tie‐ying Huo, Mao‐hua Zhou, You‐wei Zheng

**Affiliations:** ^1^ Division of Laboratory Medicine Guangdong Provincial People’s Hospital Guangdong Academy of Medical Sciences Guangzhou China

**Keywords:** apoptosis, CRISPR, JNK, paclitaxel, TAK1

## Abstract

Paclitaxel (PTX) has previously been used to treat tumours of various tissue origins, such as lung, breast, ovarian, prostate cancers and leukemia. PTX‐induced apoptosis is associated with p38 mitogen‐activated protein kinase (p38 MAPK), extracellular signal‐regulated kinase (ERK), nuclear factor‐kappa B (NF‐κB) and c‐Jun N‐terminal kinase or stress‐activated protein kinase (JNK/ SAPK) pathways. Transforming growth factor‐beta‐activated kinase 1 (TAK1) and TAK1‐binding protein 1 (TAB1) play an important role in cell apoptosis through the p38, ERK, NF‐κB and JNK signal transduction pathways. To investigate the role of TAK1 in PTX‐induced cell apoptosis, we treated HEK293 and 8305C cells with 0–20 µm PTX for 6, 12 or 24 h. To investigate whether TAK1 can cooperate with PTX for cancer treatment, we transfected cells with TAK1, TAB1 or control plasmid and treated them with PTX (3–10 µm) for 9–24 h. Apoptosis rates were analysed by flow cytometry (Annexin V/PI). Endogenous TAK1 and TAB1, caspase‐7 cleavage, poly ADP‐ribose polymerase (PARP) cleavage, Bcl‐xL level, phospho‐p44/42, phospho‐JNK and phospho‐p38 were detected by western blot. We show that in HEK293 and 8305C cells, PTX enhanced the endogenous TAK1/TAB1 level and induced cell apoptosis in a dose‐ and time‐dependent manner. Upon TAK1 overexpression in HEK293 cells treated with PTX, apoptosis rate, JNK phosphorylation and PARP cleavage increased contrary to heat‐shocked or untreated cells. CRISPR editing of the *tak1* gene upon PTX treatment resulted in lower phospho‐JNK and PARP cleavage levels than in cells transfected with the control or the TAK1‐ or TAB1 + TAK1‐containing plasmids. TAK1‐K63A could not induce JNK phosphorylation or PARP cleavage. We conclude that PTX induces HEK293 and 8305C cell apoptosis through the TAK1–JNK activation pathway, potentially highlighting TAK1’s role in chemosensitivity.

## Graphical abstract

Paclitaxel induces HEK293 and 8305C cell apoptosis through the Transforming growth factor‐beta‐activated kinase 1 (TAK1)–c‐Jun N‐terminal kinase (JNK) activation pathway. Paclitaxel increases endogenous TAK1 and TAK1‐binding protein 1(TAB1) levels in HEK293 and 8305C cells in a dose‐ and time‐dependent manner. TAK1 and TAB1 complex induced JNK activation through phosphorylation, which leads to inhibition of Bcl‐xL.

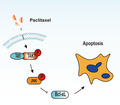

**Abbreviations**
ERK: extracellular signal‐regulated kinase
JNK/SAPK: c‐Jun N‐terminal kinase or stress‐activated protein kinase
MAP3K: mitogen‐activated protein kinase kinase kinase
NF‐κB: nuclear factor‐kappa B
PARP: poly ADP‐ribose polymerase
PTX: paclitaxel
SD: standard deviation
TAB1: TAK1‐binding protein 1
TAK1: transforming growth factor‐beta‐activated kinase 1
WB: western blot

Paclitaxel (PTX) is a taxane that is derived from the bark of the Pacific yew tree, *Taxus brevifolia*. PTX may be used alone or in combination with other chemotherapeutic agents to treat tumours of various tissue origins, such as lung [1, 2], breast [3, 4], ovarian [5], prostate cancer [6] and leukemia [7]. PTX‐induced apoptosis is associated with p38 mitogen‐activated protein kinase (MAPK) [8, 9, 10], extracellular signal‐regulated kinase (ERK) [8], nuclear factor‐kappa B (NF‐κB) [11] and c‐Jun N‐terminal kinase or stress‐activated protein kinase (JNK/SAPK) pathways [7, 8, 12, 13].

Transforming growth factor‐beta‐activated kinase 1 (TAK1) is a serine/threonine kinase of themitogen‐activated protein kinase kinase kinase (MAP3K) family [14]. As a common kinase, TAK1 plays a role in the crosstalk among a wide variety of intracellular signalling pathways, including the JNK/SAPK, p38 MAPK, ERK and NF‐κB pathways [15, 16, 17]. TAK1 regulates diverse cellular processes, such as embryonic development, cell differentiation, cell apoptosis and survival. TAB1 (TAK1‐binding protein 1) is a TAK1‐binding protein [17, 18]. TAK1 and TAB1 play an important role in cell apoptosis through the p38 [19, 20], ERK [2], NF‐κB [21, 22] and JNK [19, 23, 24] signal transduction pathways.

The intracellular signal transduction pathways related to PTX‐induced cell apoptosis overlap with those associated with TAK1. In this work, we hypothesized that TAK1 plays a role between PTX and downstream signalling pathways, and investigated the signal transduction pathway that is activated by PTX and TAK1.

As a chemotherapeutic drug, PTX may produce resistance. One way to improve therapeutic effects and reduce drug dosage and drug resistance is via combination with other drugs. Several teams have studied methods or drugs that can synergize with PTX to promote cancer cell apoptosis, for example, plumbagin [4], diosmetin [1], Zn [6], bazedoxifene [3] and even microRNA [25]. TAK1 may have synergistic effects with PTX on cancer cells. We investigated whether TAK1 can cooperate with PTX in cancer treatment, and explored the effects and mechanisms of PTX and TAK1 in HEK293 and 8305C cells, providing a basis for further study on cancer cells.

## Materials and methods

### Plasmids and reagents

pcDNA3.1‐TAK1‐myc and pcDNA3.1‐TAB1‐myc were self‐assembled [26]. p3XFLAG‐TAK1‐K63A was obtained from Addgene (http://www.addgene.org), whereas PTX was purchased from MedChem Express USA (33069624; MBCHEM; Monmouth Junction, NJ, USA). HBAD, HBAD‐TAB1‐3XFlag and HBAD‐TAK1‐3XFlag were purchased from Hanbio Biotechnology Co., Ltd. (Shanghai, China). Antibodies purchased from Cell Signaling Technology included anti‐PARP (9542; CST; Danvers, MA, USA), anti‐phospho‐p38 (9211; CST), anti‐phospho‐JNK (9251; CST), phospho‐P44/42 (4370; CST), anti‐IκBα (9242; CST), anti‐JNK (9252; CST), anti‐p38 (9212; CST), phospho‐IκBα (9246; CST), anti‐TAK1 (5206; CST) and anti‐caspase‐7 (9492; CST). The Annexin V–FITC Apoptosis Kit was purchased from BD Biosciences (556570; BD；San Jose, CA, USA), T7 endonuclease from New England Biolabs (M0302; NEB；Ipswich, MA，USA) and Lipofectamine 3000 from Invitrogen (L3000‐015；Carlsbad, CA, USA).

### Cell culture and transfection

HEK293 and 8305C cells were cultured in a 37 °C, 5% CO_2_ incubator in 1640 medium (10% FBS and 1% antibody). Plasmid transfection was performed according to the kit instructions (Lipofectamine 3000; Invitrogen). Adenovirus transfection multiplicity of infection is 30.

### Western blot analysis

A six‐well plate was used for cell culture and transfection. The number of cells per well was about 1 × 10^6^, and 40–50 µL protein lysate solution was used for lysis. Protein concentration was measured by the Coomassie Brilliant Blue method. Equal amounts of protein were resolved on 10–12% polyacrylamide/bis‐acrylamide gels at 80 V and transferred to poly(vinylidene difluoride) blotting membranes. Blocking was performed with 5% skimmed‐milk powder in 1× TBST for 1 h, and the primary antibody was incubated with the membranes overnight. After washing the membranes with TBST three times, HRP‐conjugated secondary antibodies were added, and the membranes were incubated at room temperature for 1 h. The membranes were washed once again with TBST three times, and chemiluminescence development was detected.

### Cell apoptosis and detection of caspase‐3 cleavage

Cells were collected and washed once with PBS (RCF 335 ***g***, 5 min). Filtered cells received 100 µL of 1× binding buffer, 5 µL Annexin V–FITC and 5 µL PI. Cells were mixed and incubated at room temperature for 15 min, and then an additional 100 µL of 1× binding buffer was added. Flow cytometry was used to analyse the apoptosis and necrosis rates. Cells were collected and permeabilized. Cleaved Caspase‐3 (Asp175) (D3E9) Rabbit mAb (Alexa Fluor 488 conjugate) (9603; CST) was used to detect caspase‐3 cleavage.

### Construction of a Cas9/CRISPR plasmid and verification of its editing effect

TAK1 (NM_145333.2) gene sequences were obtained from National Center for Biotechnology Information GenBank. Cas9/CRISPR target sites were selected to design a single‐stranded DNA fragment, TAK1 (ACCCAGCGCTAATTCA), which annealed to form a double‐stranded sequence that was inserted into the eCRISPR plasmid [27]. Sequencing results proved that the plasmid was successfully constructed. The *tak1*eCRISPR plasmid was transfected into HEK293 cells (2 µg per well, 12‐well plate), and puromycin was added 24 h later (2 µg·mL^−1^); screening was performed for 7–10 days. Cells that had not been transfected were killed, and cells in which editing had successfully occurred formed clones. The latter were collected, and cellular genomic DNA was extracted. DNA from the editing site was amplified by PCR and sequenced. The PCR products were denatured and annealed into double strands. Identification with T7 endonuclease showed that the mutant DNA was cut into short fragments, whereas the unedited DNA did not produce any cleavage bands.

### Statistical analysis

All *in vitro* data are representative of at least three independent experiments. Student’s *t*‐test was used for statistical analysis. A *P* value <0.05 was considered statistically significant.

## Results

### PTX induces HEK293 cell apoptosis in a dose‐ and time‐dependent manner, endogenous TAK1 and TAB1 levels increased, PARP shear and caspase‐7 cleavage increased simultaneously

**Fig. 1:**
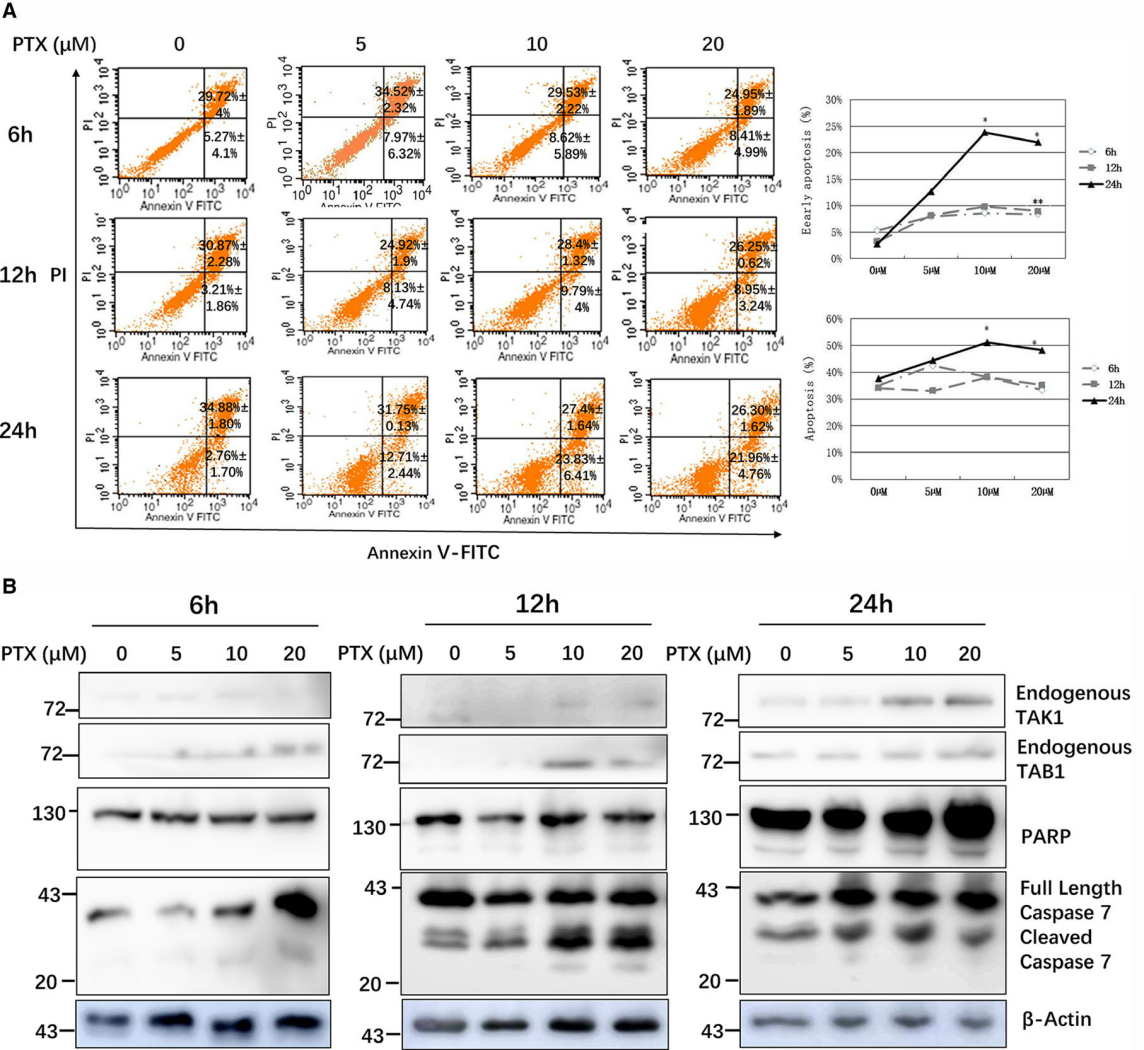
PTX induced HEK293 cell apoptosis in a dose‐ and time‐dependent manner. (A) HEK293 cells were exposed to 0, 5, 10 or 20 µm PTX for 6, 12 and 24 h, and the apoptosis rate was analysed by flow cytometry (Annexin V/PI). Results are expressed as means ± standard deviation (SD) and are representative of three independent experiments (*n* = 3); statistical analysis was performed with Student’s *t*‐test, *P < 0.05, **P < 0.01. (B) HEK293 cells were exposed to 0, 5, 10 or 20 µm PTX for 6, 12 and 24 h; endogenous TAK1 and TAB1, caspase‐7 fragments and PARP fragments were detected by WB analysis.

HEK293 cells were exposed to 0, 5, 10 or 20 µm PTX for 6, 12 and 24 h, and the apoptosis rate was analysed by flow cytometry (Annexin V/PI). The apoptosis rate increased with PTX treatment in a dose‐dependent manner, especially in the 24‐h, 10 and 20 µm wells (*P* < 0.05, *n* = 3) (Fig. 1A). At the same time, the endogenous TAK1 and TAB1 levels, caspase‐7 cleavage and PARP shear were enhanced, similarly to the apoptosis rate (Fig. 1B). These results suggested that PTX induced HEK293 cell apoptosis in a dose‐ and time‐dependent manner, increased endogenous TAK1 and TAB1 level, and enhanced caspase‐7 and PARP cleavage. TAK1/TAB1 may be placed between PTX and downstream signalling pathways that induce cell apoptosis.

### PTX and TAK1/TAB1 overexpression induces HEK293 cell apoptosis

**Fig. 2:**
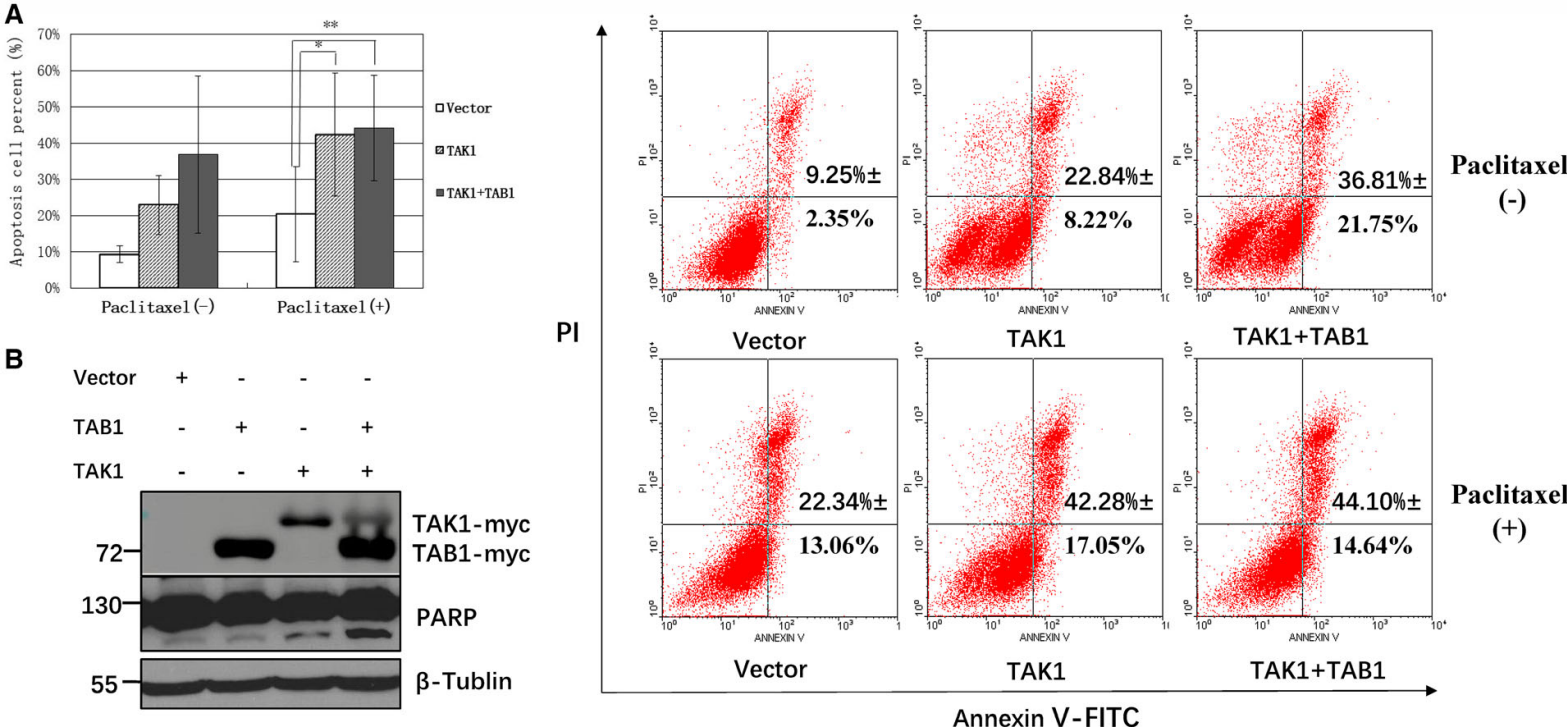
PTX treatment (3 µm, 24 h) and TAK1/TAB1 overexpression induce HEK293 cell apoptosis and PARP cleavage. (A) HEK293 cells were transfected with the control, p‐TAK1‐myc or p‐TAK1‐myc + p‐TAB1‐myc plasmid, and 48 h later, the cells were divided into two groups: untreated and treated with PTX (3 µm, 24 h). The apoptosis rate was detected by flow cytometry. In the untreated group, the apoptosis rate in the TAK1 and TAB1 + TAK1 wells increased compared with that in the control well, but there was no significant difference. In the PTX‐treated group, the apoptosis rate in the TAK1 (*P* < 0.05) and TAB1 + TAK1 (*P* < 0.01) wells increased significantly compared with that in the control well. Results are expressed as means ± SD and are representative of three independent experiments (*n* = 3); statistical analysis was performed with Student’s *t*‐test, *P < 0.05, **P < 0.01. (B) HEK293 cells were transfected with the control, p‐TAB1‐myc, p‐TAK1‐myc or p‐TAK1‐myc + p‐TAB1‐myc plasmid. PTX (3 µm) was added 48 h later, and PARP was detected by WB analysis 24 h later. PARP cleavage in the TAK1 and TAB1 + TAK1 wells was significantly enhanced.

HEK293 cells were transfected with a control vector (pcDNA3.1) and with a eukaryotic expression plasmid containing pcDNA3.1‐TAK1‐myc or pcDNA3.1‐TAK1‐myc + pcDNA3.1‐TAB1‐myc. Forty‐eight hours later, the cells were divided into untreated and PTX‐treated groups (3 µm, 24 h). Flow cytometry was used to detect the apoptosis rate. In the PTX‐treated group, the apoptosis rate in the TAK1 (*n* = 3, *P* < 0.05) and TAB1 + TAK1 (*n* = 3, *P* < 0.01) wells was significantly higher than that in the control vector well (Fig. 2A). PARP cleavage in cells transfected with TAK1 or with TAB1 + TAK1 significantly increased after 24 h of PTX (3 µm) treatment (Fig. 2B). In combination with overexpression of TAK1, PTX induced HEK293 cell apoptosis, and the dosage was reduced to 3 µm(24 h).

### PTX and TAK1 induce HEK293 cell apoptosis through the JNK pathway

**Fig. 3:**
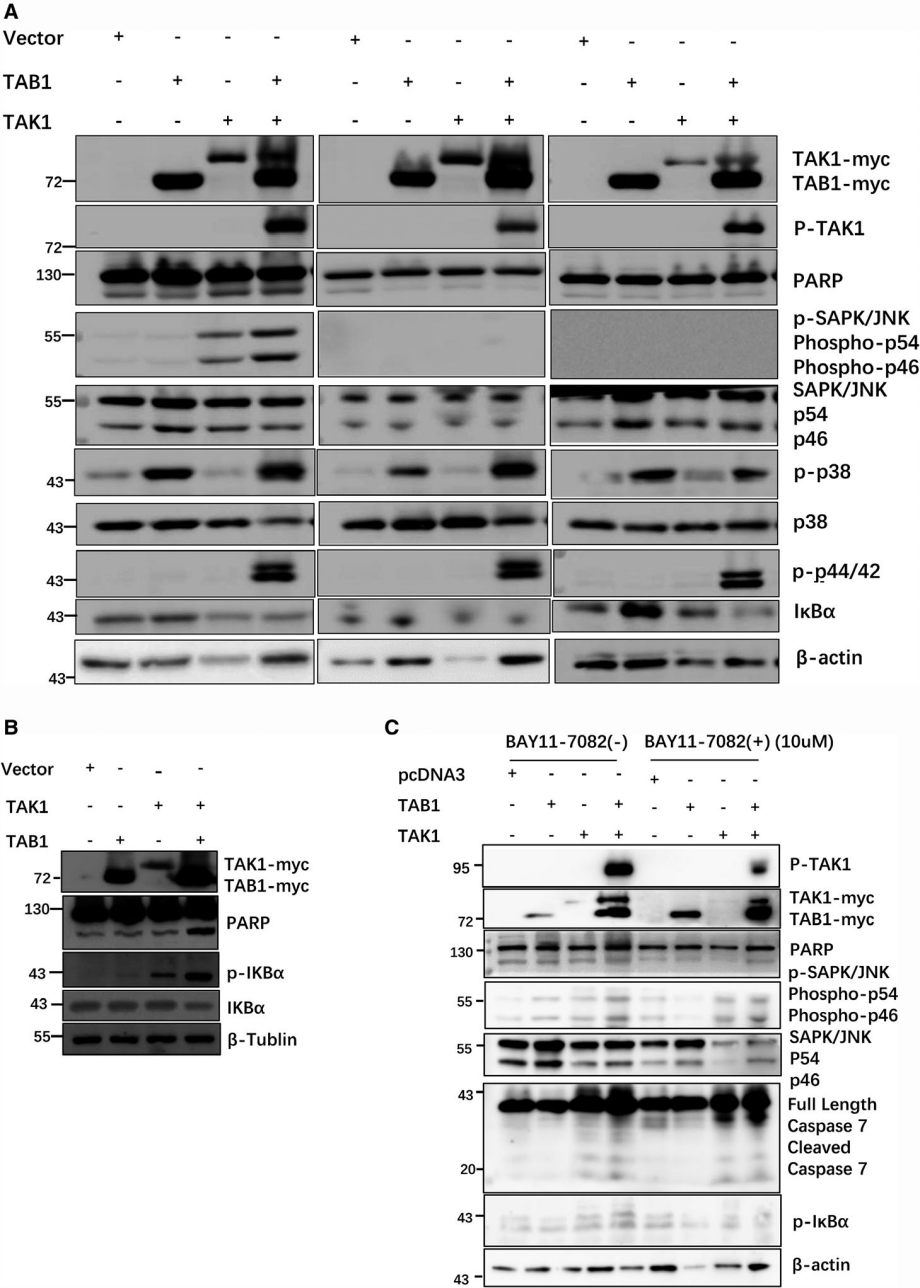
Effects of TAK1/TAB1 on PARP cleavage and the signalling pathway of HEK293 cells in the PTX, heat shock and untreated groups. (A) HEK293 cells were transfected with the control, p‐TAB1‐myc, p‐TAK1‐myc or p‐TAK1‐myc + p‐TAB1‐myc plasmid, and 48 h later, the cells were divided into three groups: PTX (10 µm, 9 h) (left), untreated (middle) and heat shock (44 °C, 10 min) (right). PARP cleavage, phospho‐JNK, phospho‐p38, phospho‐p44/42 and IκBα degradation were detected. Phospho‐JNK levels in the TAK1 and TAK1 + TAB1 wells in the PTX group significantly increased, whereas phospho‐p38, phospho‐p44/42 and IκBα degradation in this group did not differ from those in the untreated and heat shock groups. (B) Effect of TAK1/TAB1 combined with PTX (10 µm, 12 h) on HEK293 cell apoptosis. HEK293 cells were transfected with the control vector, p‐TAB1‐myc, p‐TAK1‐myc or p‐TAK1‐myc + p‐TAB1‐myc. PARP cleavage and phospho‐IκBα were detected by WB analysis, and their levels were significantly enhanced in the TAK1 and TAB1 + TAK1 overexpression wells after PTX treatment. (C) Effects of BAY11‐7082 on PARP cleavage and JNK phosphorylation in HEK293 cells. HEK293 cells were transfected with the control, p‐TAB1‐myc, p‐TAK1‐myc or p‐TAK1‐myc + p‐TAB1‐myc plasmid. Forty‐eight hours later, the cells were divided into two groups: PTX (10 µm, 12 h) group and PTX (10 µm, 12 h) + BAY11‐7082 (10 μM) group. PARP cleavage, phospho‐JNK and caspase‐7 cleavage were detected by WB analysis. In the PTX group, the phospho‐JNK band intensity and PARP cleavage in the TAK1 + TAB1 well increased as before. In contrast, in the PTX + BAY11‐7082 group, phospho‐JNK levels and PARP cleavage still increased in the TAK1 overexpression well. Caspase‐7 cleavage changed in a manner similar to PARP cleavage and phospho‐JNK levels.

HEK293 cells were transfected with the control vector, p‐TAB1‐myc, p‐TAK1‐myc or p‐TAK1‐myc + p‐TAB1‐myc. The cells were divided into three groups: PTX (10 µm, 9 h) group (Fig. 3A, left), untreated group (Fig. 3A, middle) and heat shock (44 °C, 10 min) group (Fig. 3A, right). Western blot (WB) analysis was used to detect PARP cleavage, phosphorylated JNK, phosphorylated p38, phosphorylated p44/42 and IκBα degradation. PARP cleavage and phosphorylated JNK levels were significantly increased in the TAK1 and TAK1 + TAB1 wells of the PTX group (Fig. 3A, left). The amounts of phosphorylated p38, phosphorylated p44/42 and IκBα degradation were similar to those in the untreated (Fig. 3A, middle) and heat shock groups (Fig. 3A, right). These results suggested that PTX‐TAK1 induces HEK293 cell apoptosis mainly through the JNK pathway.

As a key protein kinase in the NF‐κB signal transduction pathways, TAK1 overexpression enhanced IκBα phosphorylation (Fig. 3B). BAY11‐7082, an NF‐κB inhibitor that inhibited IκBα phosphorylation, was used to verify that JNK was the main pathway by which PTX‐TAK1 affected HEK293 cell apoptosis. BAY11‐7082 (10 µm) inhibited IκBα phosphorylation and partly affected the phosphorylation of TAK1. However, compared with the vector well, JNK phosphorylation and PARP cleavage still increased in the TAK1 + TAB1 overexpression well of the BAY11‐7082 + PTX group, and caspase‐7 cleavage changed in the same manner (Fig. 3C). These results suggest that TAK1 and PTX induce HEK293 cell apoptosis mainly through the JNK pathway.

### With TAK1/TAB1 overexpression, PTX promoted HEK293 cells apoptosis more than PTX treated alone, and *tak1* gene editing confirmed that PTX–TAK1 induces HEK293 cell apoptosis through the JNK pathway

**Fig. 4:**
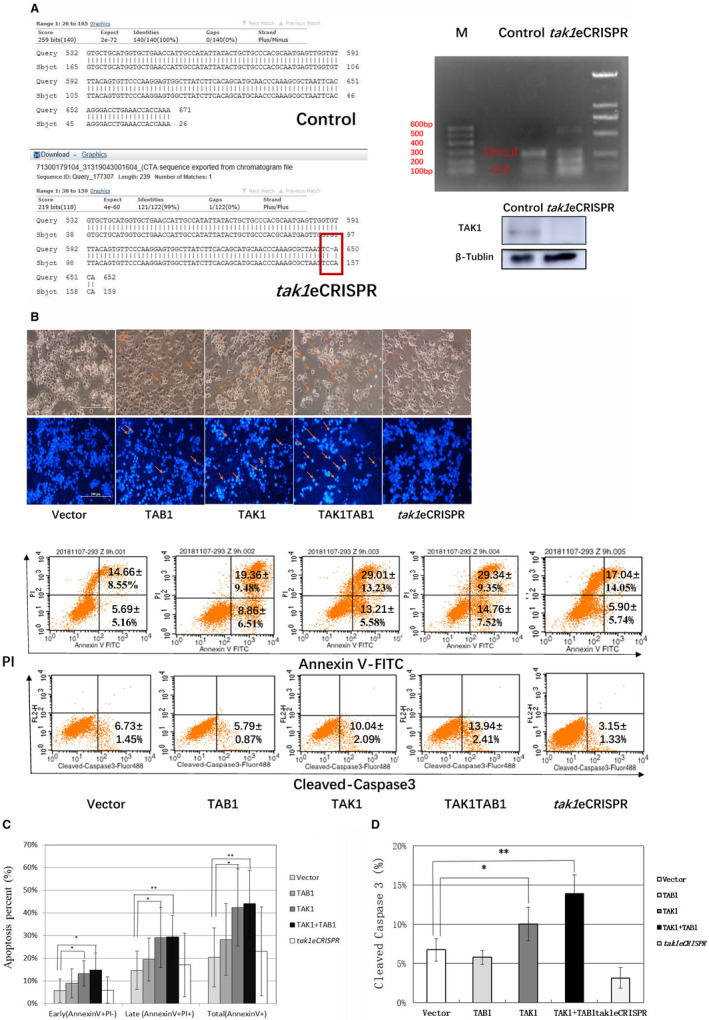
Effects of *tak1*eCRISPR combined with PTX (10 µm, 9 and 12 h) on cell morphology, cell apoptosis and caspase‐3 cleavage in HEK293 cells. (A) Validation of the gene‐editing effect of the *tak1*eCRISPR plasmid in HEK293 cells. The *tak1*eCRISPR plasmid was transfected into HEK293 cells, and puromycin was added 24 h later for a screening period of 7–10 days. Control cells did not receive the plasmid. The edited cells formed clones, which were collected and subjected to genomic DNA extraction. The editing site sequence was amplified by PCR, the fragment size was detected by electrophoresis and the PCR product was recovered from the gel. The PCR product and GenBank NM_145331 sequences were compared using blast, and the results showed that a cytosine base was inserted after gene editing, resulting in a frameshift mutation (left). Gel‐recovered DNA fragments were annealed. T7 endonuclease I digestion (37 °C, 30 min) showed that the *tak1* gene fragment (uncut, 302 bp) was cut into two short fragments (cut, approximately 100 and 200 bp), while the DNA that did not undergo gene editing did not produce cleavage bands (control) (top right). TAK1 protein expression in the control and in the *tak1* gene‐edited HEK293 cells was shown by WB analysis (bottom right). (B) Effects of TAK1/TAB1 combined with PTX (10 µm, 12 h) on HEK293 cell morphology. HEK293 cells were transfected with the control, p‐TAB1‐myc, p‐TAK1‐myc, p‐TAK1‐myc + p‐TAB1‐myc or *tak1*eCRISPR plasmid. Forty‐eight hours later, PTX (10 µm, 12 h) was added. Compared with the control, the cell density in the TAK1 well and especially in the TAB1 + TAK1 well decreased, the cells became round and the nuclei shrank. The cell morphology of the *tak1*eCRISPR well was similar to that of the vector well. Scale bars: 200 µm. Red arrows indicate cells became round and cell nuclei shrank. (C) Effect of TAK1/TAB1 combined with PTX (10 µm, 9 h) on HEK293 cell apoptosis. HEK293 cells were transfected with the control, p‐TAB1‐myc, p‐TAK1‐myc, p‐TAK1‐myc + p‐TAB1‐myc or *tak1*eCRISPR plasmid. The apoptosis rate was detected by flow cytometry. After PTX was added (10 µm, 9 h), the apoptosis rates in the TAK1 and TAB1 + TAK1 wells increased significantly in comparison with that in the pcDNA3.1 vector well. Results are expressed as means ± SD and are representative of seven independent experiments (*n = 7*); statistical analysis was performed with Student’s *t*‐test, *P < 0.05, **P < 0.01. (D) Effect of TAK1/TAB1 combined with PTX (10 µm, 9 h) on caspase‐3 cleavage. HEK293 cells were transfected with the control, p‐TAB1‐myc, p‐TAK1‐myc, p‐TAK1‐myc + p‐TAB1‐myc or *tak1*eCRISPR plasmid. The caspase‐3 cleavage rate was detected by flow cytometry. After PTX was added (10 µm, 9 h), the caspase‐3 cleavage rates in the TAK1 and TAB1 + TAK1 wells increased significantly in comparison with that in the vector well. Results are expressed as means ± SD and are representative of five independent experiments (*n* = 5); statistical analysis was performed with Student’s *t*‐test, *P < 0.05, **P < 0.01.

The *tak1*eCRISPR plasmid was constructed and verified by sequencing. The plasmid was transfected into HEK293 cells, which were screened with puromycin. Genomic DNA was extracted, and the edited fragment was amplified. Comparison between the PCR product sequence and the GenBank NM_145331 sequences through a blast search showed that a cytosine base was inserted after gene editing, resulting in a frameshift mutation (Fig. 4A, left). After T7 endonuclease digestion, the edited DNA fragment was cut into short fragments, which proved that the plasmid was effective for *tak1* gene editing (Fig. 4A, top right). TAK1 protein expression decreased in *tak1* gene‐edited HEK293 cells (Fig. 4A, bottom right).

**Fig. 5:**
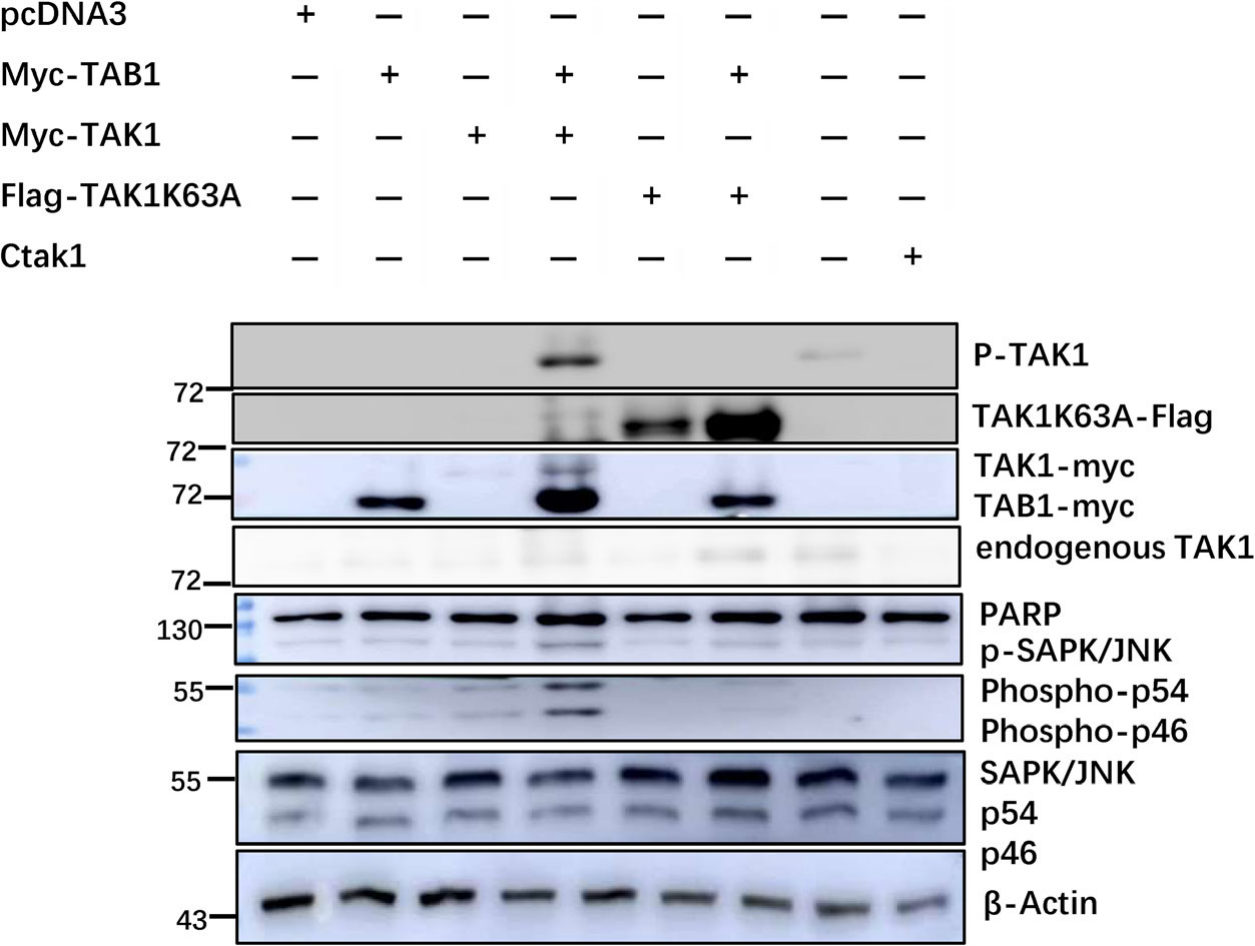
Effects of *tak1*eCRISPR and TAK1‐K63A combined with PTX (10 µm, 12 h) on PARP cleavage and JNK phosphorylation in HEK293 cells. HEK293 cells were transfected with the control, p‐TAB1‐myc, p‐TAK1‐myc, p‐TAK1‐myc + p‐TAB1‐myc, p3XFLAG‐TAK1‐K63A, p‐TAK1‐K63A + p‐TAB1‐myc or *tak1*eCRISPR plasmid. After PTX (10 µm, 12 h) treatment, PARP cleavage, phospho‐TAK1 and phospho‐JNK were detected by WB analysis. In the TAK1 and TAK1 + TAB1 wells, phospho‐JNK band intensity and PARP cleavage greatly increased. In the p3XFLAG‐TAK1‐K63A well, PARP cleavage, phospho‐TAK1 band intensity and phospho‐JNK band intensity were weakened in comparison with those in the TAK1 and TAK1 + TAB1 wells. In the *tak1*eCRISPR well, the level of endogenous TAK1 was reduced, and PARP cleavage, phospho‐TAK1 band intensity and phospho‐JNK band intensity were weakened in comparison with those in the vector, TAK1 and TAK1 + TAB1 wells.

HEK293 cells were transfected with the control, TAB1‐myc, TAK1‐myc + TAB1‐myc or *tak1*eCRISPR plasmid. After PTX treatment (10 µm, 12 h), 4,6‐diamino‐2‐phenyl indole (DAPI) staining was performed. Compared with the control, the cell density in the TAK1 and TAB1 + TAK1 wells decreased, the cells became round and the nuclei shrank. In the TAB1 + TAK1 well, this effect was more evident (Fig. 4B). Cell apoptosis and caspase‐3 cleavage were detected by flow cytometry. The apoptosis rates in the TAK1 (*n* = 7, *P < 0.05) well and especially in the TAB1 + TAK1 overexpression well (*n* = 7, **P < 0.01) were significantly higher than that in the control vector well (Fig. 4C). The caspase‐3 cleavage in the TAK1 (*n* = 5, *P < 0.05) well and especially in the TAB1 + TAK1 overexpression well (*n* = 5, **P < 0.01) was higher than that in the control vector well (Fig. 4D). WB assays showed that PARP cleavage and phospho‐JNK levels increased in the TAK1 and TAK1 + TAB1 overexpression wells, whereas in the *tak1*eCRISPR well, the level of endogenous TAK1 was reduced, and the levels of phospho‐JNK and PARP cleavage were lower than those in the control vector, TAK1 and TAB1 + TAK1 wells (Fig. 5), confirming that TAK1 is necessary for PTX‐mediated induction of HEK293 cell apoptosis through the JNK pathway.

### PTX and TAK1 overexpression induce HEK293 cell apoptosis, which is associated with TAK1 phosphorylation

Transfection of P3XFLAG‐TAK1‐K63A into HEK293 cells, either alone or cotransfected with TAB1, did not lead to TAK1‐K63A phosphorylation. Compared with the wild‐type TAK1 + TAB1 overexpression well, PARP cleavage and JNK phosphorylation were not significantly induced in the TAK1‐K63A and TAK1‐K63A + TAB1 overexpression wells. It is suggested that TAK1 overexpression induces HEK293 cell apoptosis, which is related to TAK1 phosphorylation. When TAB1 is also overexpressed, the effect is more significant (Fig. 5).

### TAK1 overexpression reduced the dose and action time of PTX, and PTX and TAK1–JNK promoted Bcl‐xL inhibition in HEK293 cells

**Fig. 6:**
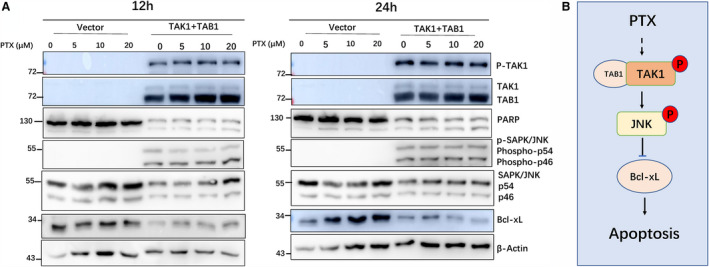
Effects of TAK1 and TAB1 with PTX (0, 5, 10, 20 µm) on PARP cleavage and JNK phosphorylation in HEK293 cells. (A) HEK293 cells were transfected with the control, p‐TAK1‐myc + p‐TAB1‐myc plasmid. After PTX (0, 5, 10 and 20 µm; 12 and 24 h) treatment, PARP cleavage, phospho‐TAK1 and phospho‐JNK were detected by WB analysis. In the TAK1 + TAB1 wells, phospho‐JNK band intensity and PARP cleavage greatly increased, and Bcl‐xL inhibition increased. (B) PTX induces apoptosis through the TAK1–JNK–Bcl‐xL pathway. PTX increases endogenous TAK1 and TAB1 level in HEK293 in a dose‐ and time‐dependent manner. The TAB1 and TAK1 complex induced JNK activation through phosphorylation, which leads to Bcl‐xL inhibition.

HEK293 cells were exposed to 0, 5, 10 or 20 µm PTX for 12 and 24 h, with or without p‐TAK1‐myc + p‐TAB1‐myc plasmid transfected. In the 12‐ and 24‐h PTX treatment group, PARP cleavage enhanced more in the TAK1‐myc + p‐TAB1‐myc plasmid‐transfected wells than the only PTX‐treated well. TAK1 + TAB1 overexpression reduces the dose and action time of PTX. Bcl‐xL is one member of the antiapoptotic Bcl2 family; TAK1 + TAB1 overexpression induced JNK phosphorylation and Bcl‐xL inhibition more than the only PTX‐treated group (Fig. 6A). These results suggested that PTX and TAK1–JNK induced HEK293 cell apoptosis in a dose‐ and time‐dependent manner, and Bcl‐xL could be placed downstream. PTX induces cell apoptosis through the TAK1–JNK–Bcl‐xL pathway (Fig. 6B).

### PTX–TAK1–JNK–Bcl‐xL induced apoptosis in 8305C cells, as well as in HEK293 cells

**Fig. 7:**
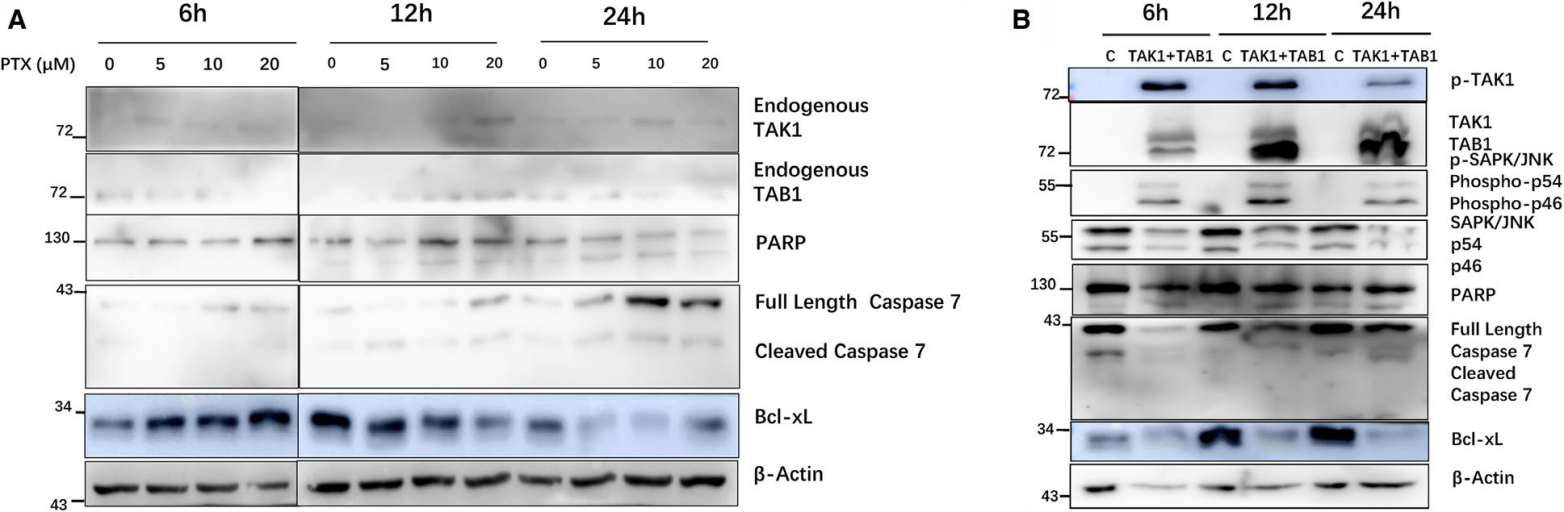
PTX induced 8305C cell apoptosis in a dose‐ and time‐dependent manner. (A) 8305C cells were exposed to 0, 5, 10 or 20 µm PTX for 6, 12 and 24 h, and endogenous TAK1 and TAB1, caspase‐7 fragments, PARP fragments and Bcl‐xL level were detected by WB analysis. (B) 8305C cells were transfected with HBAD, HBAD‐TAB1‐3XFlag and HBAD‐TAK1‐3XFlag, and 48 h later were exposed to 10 µm PTX for 6, 12 and 24 h. p‐TAK1, TAK1 and TAB1, PARP fragments, caspase‐7 fragments and Bcl‐xL level were detected by WB analysis.

HEK293 cell is a good model cell; however, it is not a tumour cell. To further verify whether PTX–TAK1 has the same mechanism in tumour cells as in HEK293 cells, we exposed thyroid tumour cell 8305C to 0, 5, 10 or 20 µm PTX for 6, 12 and 24 h. Along with PTX, in a dose‐ and time‐dependent manner, the endogenous TAK1 and TAB1 levels, caspase‐7 cleavage and PARP shear were enhanced, and Bcl‐xL level was inhibited. These results suggested that PTX induced 8305C cells apoptosis the same as HEK293 cells (Fig. 7A).

Later, 8305C cells were transfected with the control, TAK1‐flag + TAB1‐flag adenovirus. After PTX treatment (10 µm, 6, 12 and 24 h), WB assays showed that PARP cleavage and caspase‐7 cleavage were enhanced, Bcl‐xL level was inhibited and phospho‐JNK levels were increased in the TAK1 + TAB1 overexpression well more than in the vector well, confirming that TAK1 is necessary for PTX‐mediated induction of cell apoptosis through the JNK pathway. TAK1 could be placed between PTX and downstream signalling pathways that induce cell apoptosis in 8305C cells (Fig. 7B).

## Discussion

This study explored the effects and mechanisms of TAK1 in PTX‐induced cell apoptosis. Primarily, we found that PTX induces HEK293 cell apoptosis, increasing endogenous TAK1 and TAB1 levels and enhancing caspase‐7 and PARP cleavage in a dose‐ and time‐dependent manner. PTX and TAK1 caused HEK293 cells to become round and their nuclei to shrink, as well as increase PARP cleavage and the apoptosis rate. Subsequently, the HEK293 cells were divided into PTX‐treated, heat shock and untreated groups. In the PTX group, the cell apoptosis rate and PARP cleavage in the TAK1 overexpression well were significantly higher than those in the control well; the levels of phosphorylated p38, phosphorylated p44/42 and IκBα degradation in the PTX group were similar to those in the untreated and heat shock groups, whereas JNK phosphorylation greatly increased. The NF‐κB inhibitor BAY11‐7082 inhibits IκBα phosphorylation but could not inhibit the PARP shear and JNK phosphorylation induced by PTX and TAK1 overexpression. These results confirmed that PTX and TAK1 induced HEK293 cell apoptosis mainly through the JNK pathway. Then, *tak1*eCRISPR was used to edit the *tak1* gene. The phospho‐JNK band intensity and PARP cleavage were lower than those in the control vector and TAK1 overexpression wells, confirming that PTX‐TAK1 induced HEK293 cell apoptosis through the JNK pathway. Later, overexpressed TAK1‐K63A could not be phosphorylated and could not significantly induce PARP cleavage and JNK phosphorylation in HEK293 cells, suggesting that the induction of HEK293 cell apoptosis by TAK1 through the JNK signalling pathway is related to TAK1 phosphorylation. Finally, PTX–TAK1 induces HEK293 cells apoptosis through JNK phosphorylation and Bcl‐xL inhibition. The PTX–TAK1–JNK–Bcl‐xL pathway induced apoptosis in 8305C cells, as well as in HEK293 cells.

### TAK1 could be placed between PTX and downstream signalling pathways

Several research teams have reported that PTX‐induced apoptosis is associated with p38 [8, 9, 10], JNK [7, 8, 12, 13], ERK [8] and NF‐κB [11], and TAK1 is a key kinase in these signal transduction pathways [15, 16, 17, 18, 20, 21, 22]. PTX mediated dose‐ and time‐dependent induction of apoptosis and an increase in endogenous TAK1 and TAB1 levels. It is suggested that TAK1 and TAB1 could be located between PTX and downstream signalling pathways, such as the p38, JNK and ERK pathways, and play a role in the pathway of PTX‐induced apoptosis.

### With PTX treatment, TAK1 overexpression promoted HEK293 cell apoptosis

Many groups have proposed evidence for the role of TAK1 in the promotion of apoptosis: TAK1 mediates renal tubular epithelial cell apoptosis via the p38 signalling pathway [20], and TAK1 overexpression and Sef‐S (similar expression to *FGF* genes, IL‐17RD) enhance UV‐induced HEK293T cell apoptosis [19]. In this work, PTX mediated dose‐ and time‐dependent induction of endogenous TAK1 and TAB1 levels and, together with TAK1 overexpression, promoted HEK293 cells apoptosis more than PTX treated alone, suggesting that PTX induces apoptosis through the TAK1 activation pathway.

### With PTX treatment, TAK1 induced HEK293 cell apoptosis through the JNK pathway

In mammalian cells, JNK plays a key role in the development and progression of cancer. Evidence has unequivocally revealed the role of JNK in cell apoptosis, and this protein has been traditionally associated with a resistance phenotype against genotoxic agents such as chemotherapeutic drugs. JNK is a positive regulator of genotoxic stress‐induced apoptosis [28, 29]. Other teams have reported that melittin promotes the apoptosis of hepatocellular carcinoma cells by activating the CaMKII–TAK1–JNK/p38 pathway [23]. The activation of TAK1 by calcium signals is, in general, an attractive possibility for explaining the almost ubiquitous phenomenon of sustained JNK activation during apoptosis [28]. In our study, to identify through which signal pathway PTX and TAK1 affect cell apoptosis, JNK, p38, ERK or NF‐κB signal pathway, we used TAK1/TAB1 overexpression, and HEK293 cells were divided into PTX‐treated, heat shock and untreated groups. The apoptosis rate, PARP cleavage and JNK phosphorylation in TAK1‐overexpressing cells greatly increased only in the PTX‐treated group. PTX induced apoptosis through JNK [7, 8]. The JNK pathway is the common mode of action for TAK1 and PTX. TAK1 is placed between PTX and JNK. It is suggested that PTX induces HEK293 cell apoptosis through the TAK1–JNK pathway.

### *tak1* was edited to confirm that PTX induced HEK293 cell apoptosis through the TAK1–JNK pathway

Research on the effect of TAK1 inhibitors on apoptosis has also been a hot topic in recent years. It has been reported that a TAK1 inhibitor promotes the apoptosis of cervical, ovarian and lung cancers, as well as lymphoma cells [21, 30, 31]. As a common protein kinase in multiple signal transduction pathways in cells from different tissues, TAK1 or its inhibitor may activate different signalling pathways, and this will be the focus of our follow‐up study. In this study, the plasmid *tak1*eCRISPR was used to edit the *tak1* gene and reduce endogenous expression of TAK1 in a more direct manner than using a chemical inhibitor. After *tak1* gene editing, the levels of phospho‐JNK and PARP cleavage were lower than those in the control vector and the TAK1‐overexpressing wells. PTX treatment could not induce apoptosis in cells harbouring edited TAK1, confirming that PTX induced HEK293 cell apoptosis through the TAK1–JNK pathway.

### PTX and TAK1 induce HEK293 cell apoptosis, and this effect is associated with TAK1 phosphorylation

In *Drosophila*, Rbf1–Rac1–dTAK1(JNKKK)–dMekk1(JNKK)–JNK signalling pathways are required for induction of apoptosis [29]. In mammalian cells, JNK regulates apoptosis through two distinct mechanisms: one is the TAK1–JNK–c‐Jun–ATF‐2–AP‐1–Fas/FasL signalling pathway, and the other is the TAK1–JNK–Bcl‐2/Bcl‐xL–cytochrome *c* signalling pathway [32, 33]. TAK1 is the common upstream MAP3K, and JNK phosphorylation induced by TAK1 is the key step in cell apoptosis. In our study, TAK1–K63A could not be phosphorylated either alone or when cotransfected with TAB1, and could not significantly induce JNK phosphorylation or PARP cleavage. This finding suggests that TAK1 phosphorylation is important for the apoptosis induced by TAK1 overexpression combined with chemotherapeutic drugs.

### TAK1 overexpression reduces the dose and action time of PTX

PTX and TAK1 overexpression induce cell apoptosis. When PTX was used alone, 20 µm and 24 h were the most significant dose and action time for induction of apoptosis, respectively (Fig. 1). When combined with TAK1 overexpression, PTX at 3 µm for 24 h (Fig. 2) or 10 µm for 9–12 h (Fig. 4) could clearly induce cell apoptosis, which indicated that TAK1 overexpression could reduce the dose and action time of PTX.

### PTX‐induced TAK1–JNK–Bcl‐xL signalling pathway exists in HEK293 and 8305C cells

As a member of the antiapoptotic Bcl2 family, Bcl‐xL is an antiapoptotic protein that prevents caspase activation. JNK‐mediated Bcl‐xL phosphorylation and inhibition are important for apoptosis in cells with Bcl‐xL expression [34]. In our study, for HEK293 and 8305C cells, PTX induces Bcl‐xL inhibition and cell apoptosis. PTX and TAK1 overexpression induce JNK phosphorylation and Bcl‐xL inhibition more than PTX alone, and the PTX‐induced TAK1–JNK–Bcl‐xL signalling pathway constitutes a complete pathway for PTX‐induced apoptosis in HEK293 and 8305C cells.

HEK293 is a good model cell, however, it is not a tumour cell. The same as in HEK293 cells, PTX‐induced endogenous TAK1 and TAB1 levels were increased, caspase‐7 and PARP cleavage were enhanced, and Bcl‐xL was inhibited in a dose‐ and time‐dependent manner in 8305C cells. PTX and TAK1–JNK induced apoptosis in 8305C cells, as well as in HEK293 cells. In HEK293 and 8305C cells, TAK1/TAB1 overexpression can reduce the dose and action time of PTX. That PTX combined with TAK1/TAB1 can be used in tumour disease treatment in the future needs to be verified in subsequent studies. This work provides a useful reference and a comparative analysis for follow‐up research.

## Conflict of interest

The authors declare no conflict of interest.

## Author contributions

Y‐WD conceived and designed the project, acquired the data of Cas9/CRISPR, analysed the data, interpreted the data and wrote the paper; ZL and SX cultured and transfected cells and acquired the data of WB; GH extracted nucleic acids from cells; YL acquired the data of flow cytometry; TH and MZ provided some equipment needed for the experiment; and YZ acquired the PCR data.

## Data Availability

No additional data are available for this article.
